# Sensors and Sensor Fusion Methodologies for Indoor Odometry: A Review

**DOI:** 10.3390/polym14102019

**Published:** 2022-05-15

**Authors:** Mengshen Yang, Xu Sun, Fuhua Jia, Adam Rushworth, Xin Dong, Sheng Zhang, Zaojun Fang, Guilin Yang, Bingjian Liu

**Affiliations:** 1 Department of Mechanical, Materials and Manufacturing Engineering, The Faculty of Science and Engineering, University of Nottingham Ningbo China, Ningbo 315100, China; mengshen.yang@nottingham.edu.cn (M.Y.); fuhua.jia@nottingham.edu.cn (F.J.); bingjian.liu@nottingham.edu.cn (B.L.); 2 Ningbo Institute of Materials Technology and Engineering, Chinese Academy of Sciences, Ningbo 315201, China; fangzaojun@nimte.ac.cn; 3 Zhejiang Key Laboratory of Robotics and Intelligent Manufacturing Equipment Technology, Ningbo 315201, China; 4 Nottingham Ningbo China Beacons of Excellence Research and Innovation Institute, University of Nottingham Ningbo China, Ningbo 315100, China; 5 Department of Mechanical, Materials and Manufacturing Engineering, University of Nottingham, Nottingham NG7 2RD, UK; xin.dong@nottingham.ac.uk; 6 Ningbo Research Institute, Zhejiang University, Ningbo 315100, China; szhang1984@zju.edu.cn

**Keywords:** self-contained localization, odometry, SLAM, polymeric sensor, state estimation, sensor fusion, IMU, LiDAR, radar, camera

## Abstract

Although Global Navigation Satellite Systems (GNSSs) generally provide adequate accuracy for outdoor localization, this is not the case for indoor environments, due to signal obstruction. Therefore, a self-contained localization scheme is beneficial under such circumstances. Modern sensors and algorithms endow moving robots with the capability to perceive their environment, and enable the deployment of novel localization schemes, such as odometry, or Simultaneous Localization and Mapping (SLAM). The former focuses on incremental localization, while the latter stores an interpretable map of the environment concurrently. In this context, this paper conducts a comprehensive review of sensor modalities, including Inertial Measurement Units (IMUs), Light Detection and Ranging (LiDAR), radio detection and ranging (radar), and cameras, as well as applications of polymers in these sensors, for indoor odometry. Furthermore, analysis and discussion of the algorithms and the fusion frameworks for pose estimation and odometry with these sensors are performed. Therefore, this paper straightens the pathway of indoor odometry from principle to application. Finally, some future prospects are discussed.

## 1. Introduction

Knowing the position of a robot is a requisite condition for conducting tasks such as autonomous navigation, obstacle avoidance, and mobile manipulation. This technology has notable economic values, and the global market of indoor Positioning, Localization, and Navigation (PLAN) is expected to reach USD 28.2 billion by 2024 [1]. The social benefit of indoor PLAN is also profound, as it may serve as a wayfinder for humans in metro stations, markets, and airports. In particular, vulnerable people—for instance, the elderly and the visually impaired—may also benefit from this technology. Although Global Navigation Satellite Systems (GNSSs) are already a mature solution for precise outdoor localization, they may quickly degrade due to satellite coverage fluctuation, multipath reflection, and variation in atmospheric conditions; such degradation is profound in indoor environments. To alleviate this effect, several approaches have been proposed, including magnetic guidance, laser guidance, Wi-Fi, Ultra-Wide Band (UWB), 5G, etc. However, these methods require the pre-installation of infrastructure such as beacons, and to change the arrangement of such a system is a tedious task. Thus, a self-contained localization system is more favorable for agents operating in such indoor environments.

During the past two decades, the self-contained odometry methodologies and Simultaneous Localization and Mapping (SLAM) technology have developed rapidly and enabled bypassing of the aforementioned problems. Odometry entails deducing the trajectory of the moving agent based on readings from observations and, with the initial position and the path of travel recorded, estimating its current position [2]. Odometry can be regarded as a thread of SLAM, where the aim is to track the position of the robot and to maintain a local map, while SLAM pursues a globally consistent trajectory and map [3]. Odometry usually serves as the front end of a SLAM system. In this review, onboard sensor systems as well as the algorithms used for mobile robots’ indoor odometry are our focus.

Materials with novel properties promote the development of odometry technologies. Magnetic materials have been utilized to build compasses for centuries, since the Earth’s geomagnetic field is able to provide an accurate and reliable reference for orientation. In the modern era, inertial navigation systems built using piezoelectric materials and electro-optic materials have emerged. In addition, photoelectric materials and electro-optic materials are applied in state-of-the-art navigation sensors, including LiDAR and cameras.

Apart from the materials mentioned above, recent advancements in materials and manufacturing technologies enable polymer-based sensors to be deployed. The first use of polymeric materials in navigation sensors can be traced back to the early 2000s [4]. Polymeric materials introduce flexibility to the sensors in terms of both mechanical structure and functionality, and reduce the cost of mass production. Such materials have been implemented in odometry sensors such as IMUs and LiDAR. Soft polymeric sensors are also ideal candidates to be embedded in soft robotics [5]. In this review, the applications of polymeric sensors for odometry are also included.

This review paper surveys the literature published in English; the search was mainly conducted in IEEE Xplore, ScienceDirect, ACM Digital Library, and arXiv. Taking IEEE Xplore as an example, the Boolean operators “AND” and “OR” were used for combining keywords as well as their synonyms. For polymeric sensors, (“polymeric” OR “polymer”) AND (“accelerometer” OR “gyroscope” OR “LiDAR” OR “Radar”) was used for searching for articles; the initial search yielded 407 papers; the papers that explicitly conceptualized or fabricated a sensor in which polymer materials played a key role—such as an actuator—were retained, while the papers that did not have a major novelty, or concerned the topic of radar-absorbing materials, were screened. For odometry, (“inertial” OR “IMU” OR “LiDAR” OR “Laser” OR “Radar” OR “visual” OR “vision”) AND (“odometry” OR “localization” OR “SLAM”) was used for searching for publications between 2015 to 2022 within journals or highly renowned conference proceedings, yielding 7821 papers. Due to the huge amount of results, only papers with more than 50 citations, or papers published after 2020 with more than 10 citations, were retained at this stage. Among the retained papers, those papers using multiple sensors, demonstrating no major novelty in terms of algorithms, focusing mainly on mapping, focused on outdoor environments, or primarily applied for mobile phones or pedestrian gait analysis were excluded. For sensor fusion, (“sensor fusion” OR “filter” OR “optimization”) AND (“odometry” OR “localization”) was used for searching for publications between 2010 and 2022 within renowned journals or conference proceedings, yielding 388 papers; those using similar sensor suites and similar fusion frameworks, using inapplicable sensors for indoor mobile robots, or focusing on outdoor environments were eliminated. After the preliminary screening, a snowballing search was conducted with the references from or to the selected papers, to check for additional papers. As a result, a total number of 252 articles was selected for review.

Several reviews have been published on odometry and SLAM. Table 1 summarizes and remarks on some of the representative works from the past five years:

To the best of our knowledge, existing reviews rarely cover sensing mechanisms, polymer-based sensors, odometry algorithms, and sensor fusion frameworks systematically in one paper. A summary table and a highlight of easy-to-use solutions are appended to the end of each section. This review paper extends previous works from three perspectives: firstly, the operating principles and advancements of sensors for odometry, including polymer-based sensors; secondly, a briefing on odometry algorithms and the taxonomy of methods based on their working principles; thirdly, a briefing and taxonomy of sensor fusion techniques for odometry. The paper is organized as follows: Section 2 reviews the operating principles of sensors for odometry, including IMU, LiDAR, radar, and camera, as well as the corresponding odometry algorithms. A summative table of representative methods is provided for each sensor. The utilization of polymeric sensors is also presented accordingly. Section 3 reviews the sensor fusion methods and their implementation in odometry. Lastly, Section 4 and Section 5 present future prospects and conclusions, respectively.

## 2. Sensors and Sensor-Based Odometry Methods

Sensors are equipped in robotic systems to mimic human sensory systems (e.g., vision, equilibrium, kinesthesia), which provide signals for perception, utilization, and decision. Onboard sensors for mobile robots can be categorized into proprioceptive sensors and exteroceptive sensors, which are sensors for monitoring internal states and the external environment, respectively. Examples of proprioceptive sensors include wheel odometers and IMUs. Examples of exteroceptive sensors include LiDAR, radar, and cameras [14].

Selectivity and sensitivity are two prominent properties of sensors. Sensitivity means the lowest power level of the signal as an input from which the sensor can decode information correctly. Selectivity means the ability to detect and decode signals in the presence of interfering signals. Advances of materials in device fabrication enable sensors with better sensitivity and selectivity to be manufactured. For example, the InGaAs/InP Single-Photon Avalanche Diode (SPAD) can detect the injection of a single photoexcited carrier. SiGe-based radar technology allows high-frequency operation, thus enabling better RF performances, and even high-resolution imaging is achievable with such sensors [15].

### 2.1. Wheel Odometer

The word “Odometry” is derived from the odometer, which is a device mounted to a wheel to measure the travel distance; it may also be referred to as a wheel encoder. There are three types of wheel encoder: the pulsed encoder, single-turn absolute encoder, and multi-turn absolute encoder. The pulsed encoder is the simplest implementation; it consists of a Hall sensor with a magnet, or has equally spaced grating to pass or reflect light. It is widely installed in drive motors for closed-loop control of the motor speed. For example, in the Wheeltec robot, a 500-pulse Hall encoder is used for motor speed feedback. The single-turn absolute encoder has two implementations: The first uses multiple Hall sensors to determine the absolute rotation angle, and has been used in the Stanford Doggo, which employs an AS5047P as the encoder. The second utilizes unequally spaced grating, such as the Gray code system, in conjunction with a light source and detection system, to determine the absolute rotation angle. The multi-turn absolute encoder usually consists of several single-turn encoders with a gear set, where the angle and the number of revolutions are recorded separately.

Several robots, such as TurtleBot and Segway RMP, use encoders for odometry. The OPS-9 uses two orthogonal wheel encoders, providing planar position information with good accuracy. Combining learning-based methods with wheel odometers can yield reasonably good odometry results. The authors of [16] tested the combination of Gaussian processes with neural networks and random variable inference on a publicly available encoder dataset. A CNN structure was employed for the neural network part, demonstrating better trajectory than the physical filters.

### 2.2. Inertial Measurement Units (IMUs)

The Inertial Measurement Unit (IMU) is a device that is used to estimate the position, velocity, and acceleration of a robot. It is generally composed of a gyroscope, an accelerometer, and a magnetometer (optional). Since these devices output the acceleration and the angular rate, other state variables—such as the velocity and the position of the robot—are obtained by integration of the measured data; thus, any drift or bias in the measurement of acceleration and angular rate will cause accumulation of errors in the estimation of velocity and position. The IMU systems discussed below are of the strapdown type rather than the stable platform (gimbal) type, due to their mechanical simplicity and compactness [17].

**Figure 1:**
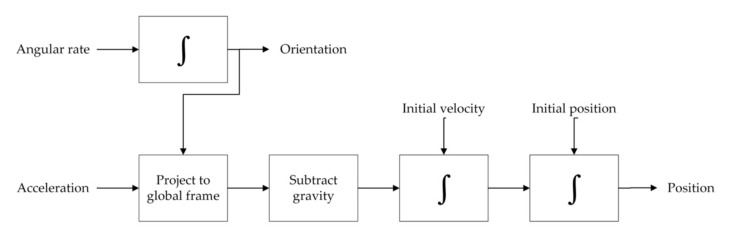
Schematic of the working principle of an IMU, redrawn from [18].

The operating principal of an IMU is shown in Figure 1. The gyroscope measures the three-axis angular rate, and estimates the relative orientation of the robot to the global frame; the accelerometer measures the acceleration, and then projects it to the global frame with the gravity vector subtracted, and the velocity and position are obtained via integration and double integration, respectively [18,19]. The IMU measurements in the local (inertial) frame are given as follows:

(1) $$\omega^{m}=\omega +b^{\omega }+\eta^{\omega }$$

(2) $$a^{m}=a+b^{a}+\eta^{a},$$

where ω and a are the true angular rate and acceleration, respectively, $b^{\omega }$ and $b^{a}$ are biases, and $\eta^{\omega }$ and $\eta^{a}$ are zero-mean white Gaussian noises. In the fixed global frame, the motion equations of the IMU are as follows:

(3) $$\dot{R}=R{\left(\omega^{m}-b^{\omega }-\eta^{\omega }\right)}_{\times }$$

(4) $$\;\dot{v}=R\left(a^{m}-b^{a}-\eta^{a}\right)+g\;$$

(5) $$\;\dot{p}=v$$

where R encodes the rotation from the local frame to the global frame, ${\left(\cdot \right)}_{\times }$ stands for the skew-symmetric matrix operator, and $g={\left[0\;0-9.81\right]}^{T}$ is the gravity vector.

**Figure 2:**
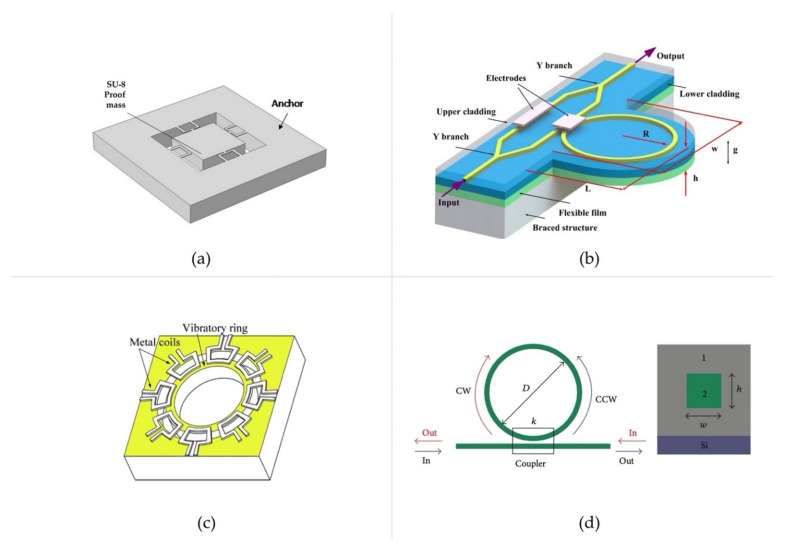
(**a**) SU-8 3-axis piezoresistive accelerometer, reprinted with permission from [20]. Copyright IEEE 2019. (**b**) Polymeric Fano-resonator-based accelerometer, reprinted with permission from [21].Copyright The Optical Society2016. (**c**) Polymeric vibratory ring-type MEMS gyroscope, reprinted with permission from [27]. Copyright IEEE 2008. (**d**) Polymeric ring resonator for optical gyroscope, reprinted from [26], Hindawi, 2014.

Different types of gyroscopes and accelerometers have been constructed based on different working principles. For accelerometers, there are three main types: piezoelectric accelerometers, capacitive Microelectromechanical System (MEMS) accelerometers, and piezoresistive MEMS accelerometers. The piezoelectric accelerometer works based on the piezoelectric effect—when a mechanical stress is applied to the crystal, it will generate a voltage. A piezoelectric accelerometer consists of one or more piezoelectric crystals and a proof mass (or seismic mass); the proof mass transduces the inertial force to the crystal when being accelerated, and the acceleration can be measured in the form of voltage. Common piezoelectric materials include PZT, LiNbO_3_, AlN, and ZnO. AlN with a wide bandgap (6.2 eV) has been regarded as the preferred material due to its high breakdown field, high working temperature, and low dielectric loss. Doping AlN with elemental Sc can significantly increase the piezoelectric coefficients. The capacitive MEMS accelerometer and the piezoresistive MEMS accelerometer are based on the mass-spring-damper system model, which utilizes capacitive change or resistive change to sense the deflection of the moving proof mass under acceleration. Compared to piezoelectric materials, piezoresistive materials have high sensitivity and better low-frequency response. SiC is regarded as a promising material; it has higher bandgap than Si, and has a good piezoresistive effect at high temperatures. Referring to the application of polymers in accelerometers, in [20], an SU-8-polymer-based, single-mass, three-axis, piezoresistive accelerometer was built (Figure 2a); it demonstrated better sensitivity due to the low Young’s modulus of SU-8 compared with Si, and piezoresistive materials such as ZnO nanorods were employed as sensing materials applied on the surface of U-beams to detect deformation. In [21], a polymeric Fano-resonator-based accelerometer was fabricated (Figure 2b); when being accelerated, a force was exerted on the ring, which experienced a strain, causing a phase change of the light proportional to the acceleration. This device demonstrated very high sensitivity. In [22], a PVDF-based localization and wheel–ground contact-sensing scheme was presented.

Gyroscopes also come in different types, including mechanical gyroscopes, optical gyroscopes, and MEMS gyroscopes [23]. Mechanical gyroscopes are constructed based on the principle of the conservation of angular momentum, which is the tendency of a moving object to maintain the same rotational axis at a constant rotation speed. Optical gyroscopes rely on the Sagnac effect. If a light path is rotating at a certain angular rate, by measuring the time delay between two light pulses travelling along the same light path in opposite directions, the angular rate can be calculated. Generally, there are two forms of optical gyroscope: Ring-Laser Gyroscopes (RLGs), and Fiber-Optic Gyroscopes (FOGs) [24]. MEMS gyroscopes are mostly based on the effect of Coriolis acceleration, which is the acceleration applied to a moving object at a certain velocity in a rotating frame. Such an MEMS gyroscope usually contains a vibrating part, and by detecting the Coriolis acceleration, the angular rate is obtained [24]. While most implementations today utilize MEMS-based gyroscopes for wheeled mobile robot applications due to their low cost, [25] used a calibrated FOG together with measurements from wheel encoders for wheeled robot dead-reckoning. Referring to the application of polymers in gyroscopes, in [26], a polymeric ring resonator was fabricated and applied to an optical gyroscope (Figure 2d). The coupler split the input light into two beams, and the refractive index difference between polymers 1 and 2 was 0.01, aiming at achieving an optimal coupling ratio and low propagation loss. In [27], a PDMS polymeric ring structure was fabricated to build an MEMS gyroscope (Figure 2c), where the bottom of the ring was fixed while the upper part could move freely. The eight coils serve as driving and sensing parts. This was achieved by exerting Lorentz force on the ring for harmonic vibration. When being rotated, a Coriolis force was introduced to the ring, and could be sensed by the coils in the form of electromotive force.

In more general cases, accelerometers and gyroscopes are often combined to form an IMU package for robot localization and navigation. Fusing multiple sensors together requires the consideration of sensor fusion; a more detailed discussion on sensor fusion techniques is presented in the Sensor Fusion section. The authors of [28] proposed a dynamic-model-based slip detector for a wheeled robot based on an MEMS IMU and an encoder, proving successful vehicle immobilization detection (0.35% of false detection) and accurate estimates of the robot’s velocity. The authors of [29] utilized a low-cost IMU and measurements from wheel encoders to form an extended Kalman filter (EKF) scheme for a skid-steered wheeled robot, with the “virtual” velocity measurement update based on the proposed kinematic model, demonstrating relatively accurate state estimation.

Putting multiple IMUs together may improve localization accuracy. The author of [30] used multiple MEMS IMUs mounted on a vehicle fused using a Kalman filter, which achieved a maximum of 55% improvement in positioning accuracy compared to a single IMU. The authors of [31] demonstrated a solution using multiple MEMS IMUs with at least one mounted on a wheel to replace the wheel encoder with the output of the wheel rate gyro, fused together with an EKF, reporting a mean positional drift rate of 0.69% and a heading error of 2.53 degrees.

IMU pre-integration plays a key role in the state-of-the-art robot navigation systems since, in general, IMUs have a higher sampling rate (100 Hz~1 KHz) than other navigation sensors; thus, combining serial IMU readings into a single measurement becomes desirable. This method was discussed in [32,33,34], and is transcribed here as follows:

(6) $$\Delta R_{ij}=R_{i}^{T}R_{j}$$

(7) $${\Delta v}_{ij}=R_{i}^{T}\left(v_{j}-v_{i}-g\Delta t_{ij}\right)$$

(8) $${\Delta p}_{ij}=R_{i}^{T}\left(p_{j}-p_{i}-v_{i}\Delta t_{ij}-\frac{1}{2}g\Delta t_{ij}^{2}\right).$$

In terms of currently available solutions, Xsens [35] and Microstrain [36] offer IMUs and development kits for visualization and log data, and can stream odometry data via an ROS API. This is also achievable with a low-cost IMU using Arduino [37]. For an in-depth review of modern inertial navigation systems and commercially available products, please refer to [24,38].

### 2.3. LiDAR

LiDAR is named after its working principle and function, which is Light Detection and Ranging. The authors of [39,40,41,42] conducted comprehensive reviews of the principles and applications of modern LiDAR systems. Based on its working principles, LiDAR can be categorized into Time-of-Flight (ToF) LiDAR and Frequency-Modulated Continuous-Wave (FMCW) LiDAR. ToF LiDAR measures range by comparing the elapsed time between the transmitted and received signal. It dominates the market due to its simple structure, but it suffers issues such as interference from sunlight or other LiDAR devices. FMCW LiDAR adopts the same principle as FMCW radar—the transmitted laser frequency is modulated linearly against the time, and then both the range and the velocity of the observed object can be translated from the frequency difference between the transmitted and received laser wave. One notable advantage of FMCW LiDAR is its ability to directly retrieve velocity from the measurements.

Based on the laser beam steering mechanism, LiDAR can be further categorized into mechanical LiDAR and solid-state LiDAR. Mechanical steering of the laser beam actuated by a motor is the most popular solution at present due to its large Field of View (FOV), but it usually results in a very bulky implementation, and is susceptible to distortion caused by motion. Solid-state LiDAR comes in multiple forms, including MEMS LiDAR, FLASH LiDAR, and Optical Phased Array (OPA) LiDAR. Here solid-state refers to a steering system without bulky mechanical moving parts. MEMS LiDAR consists of a micromirror embedded in a chip, the tilting angle of which is controlled by the electromagnetic force and the elastic force, resulting in a relatively small FOV (typically 20–50 degrees horizontally). Due to its compact size and low weight, MEMS LiDAR can be used for robotic applications, where size and weight requirements are stringent [43]. Ref. [44] developed and fabricated an MEMS LiDAR for robotic and autonomous driving applications. The authors of [45] presented an algorithm for such small-FOV solid-state LiDAR odometry, mitigating the problems of small FOV, irregular scanning pattern, and non-repetitive scanning by linear interpolation, and demonstrating a trajectory drift of 0.41% and an average rotational error of 1.1 degrees. FLASH LiDAR operates in a similar way to a camera using a flashlight—a single laser is spread to illuminate the area at once, and a 2D photodiode detection array is used to capture the laser’s return. Since the imaging of the scene is performed simultaneously, movement compensation of the platform is unnecessary. This method has been used for pose estimation of space robots, as demonstrated by [46,47]. The main drawbacks of FLASH LiDAR are its limited detection range (limited laser power for eye protection) and relatively narrow FOV. OPA LiDAR controls the optical wavefront by modulating the speed of light passing through each antenna; ref. [48] presents a realization and application of such a device.

**Figure 3:**
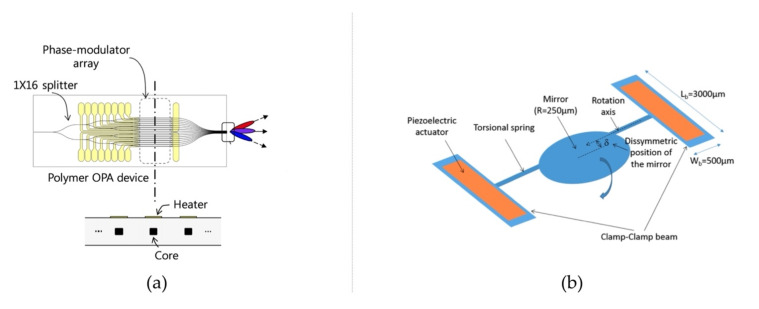
(**a**) Polymeric thermo-optic phase modulator for OPA LiDAR, reprinted from [49], Optica Publishing Group, 2020; (**b**) P(VDF-TrFE) copolymer piezoelectric actuator for MEMS LiDAR, reprinted with permission from [51]. Copyright IEEE 2018.

Polymers have been utilized for the fabrication of novel LiDAR devices. In [49,50], a polymeric thermo-optic phase modulator was fabricated and utilized for OPA LiDAR (Figure 3a), achieving good energy efficiency. In [51], piezoelectric polymer P(VDF-TrFE) copolymers were employed as actuators, introducing rotation to the micromirror of the MEMS LiDAR due to the asymmetric position of the mirror (Figure 3b). In [52], a UV-cured polymer was adopted for the fabrication of microlenses of Single-Photon Avalanche Diodes (SPADs) applied in FLASH LiDAR.

LiDAR odometry (LO) is the process of finding the transformation between consecutive LiDAR scans, by aligning the point cloud from the current scan to the reference scan. Reviews of the point cloud alignment methods for general purposes can be found in [53,54,55,56,57], and for autonomous driving in [58,59]. Based on the source of the LiDAR point cloud, both 2D [60,61] and 3D implementations have been shown. Those methods can be categorized into scan-matching methods and feature-based methods [59].

Scan-matching methods are also called fine-registration methods. Among the registration methods, the family of Iterative Closest Point (ICP) registration methods is widely adopted [53] not only for mobile robots’ SLAM problems [62], but also for object reconstruction, non-contact inspections, and surgery support. A comprehensive review of the ICP algorithms can be found in [63]. The general idea behind ICP is to iteratively find the transformation that can best align the incoming point cloud with the reference point cloud. A complete ICP algorithm should include functional blocks such as a data filter, initial transformation, associate solver, outlier filter, and error minimization. The association solver (also called match function) is utilized for pairing points from the incoming data and the reference point cloud; this process may also refer to data association, point matching, or correspondence finding, depending on the context. This matching process can have three types: feature matching (point coordinate, surface normal [64], or curvature [65]), descriptor matching (laser intensity [66]), and mixed. The finding process is often accelerated by data structures such as k-D trees [67] to find the correspondences with the shortest distance and/or similar properties.

Error minimization is the area in which where most ICP variants differ. The goal is to minimize Equation (9), where p and q are corresponding points in the reading and reference point clouds, respectively, while R and t are the rotation and translation to be resolved, respectively. The outcome is dependent on the error metric, for example, the point-to-point error metric, which takes the Euclidean distance between points as the metric, as shown in [68]; the point-to-plane error metric [69], which originates from the idea that the points are sampled from a smooth surface, and searches for the distance between a point and a plane containing the matched point; and the generalized ICP [70], which introduces a probabilistic representation of the points, and can be viewed as a plane-to-plane error metric. LiTAMIN2 [71] introduced an additional K–L divergence that evaluates the difference in the distribution shape, which can perform well even when the points for registration are relatively sparse.

(9) $${\left(q-\left(Rp+t\right)\right)}^{T}\left(q-\left(Rp+t\right)\right)$$

A major drawback of ICP is that it relies heavily on the initial guess. Therefore, ICP is susceptible to local minima [72]. To address this issue, a global method based on unique features without the need for initial assumption was developed. The authors of [68] proposed a globally optimized ICP that integrates the Branch-and-Bound (BnB) algorithm; the ICP searches the local minima, while the BnB algorithm helps it to jump out of the local minima, and together they converge to the global minima.

Another disadvantage of ICP is that it is a discrete sampling of the environment. To address the effect of uneven LiDAR points, the Normal Distribution Transform (NDT) was introduced for both 2D registration [73] and 3D registration [74]. Instead of using individual LiDAR points, the normal distributions give a piecewise smooth Gaussian probability distribution of the point cloud, thus avoiding the time-consuming nearest-neighbors search and memory-inefficient complete point cloud set storage. The NDT first equally divides the space occupied by the scan into cells, and then calculates the mean vector q and the covariance matrix C for each cell of the fixed reference scan. The goal is to find the transformation of R and t that can minimize the score function (Equation (10)) using Newton’s optimization method. Since this process iterates over all points in the incoming scan, this is called a Point-to-Distribution (P2D) NDT registration algorithm.

(10) $${\left(q-\left(Rp+t\right)\right)}^{T}C^{-1}\left(q-\left(Rp+t\right)\right)$$

If the registration is directly performed on the distribution model of both scans, then it becomes a Distribution-to-Distribution (D2D) NDT registration algorithm [75]. This method shares many similarities with generalized ICP in the distance error metric function, but performs more accurately and faster compared with the generalized ICP and the standard P2D [76]. An NDT histogram of plane orientation is also computed in this method, for better initial transformation estimation. However, in some cases it is still susceptible to local minima. The Gaussian Mixture Map (GMM) method [77,78] has similarities with the NDT methods, since they both maximize the probability of drawing the transformed scan from the reference scan, which constructs a Gaussian mixture model over the z-height of the 3D LiDAR scan, and then uses a multiresolution Branch-and-Bound search to reach global optima. The Coherent Point Drift (CPD) algorithm is also based on the GMM method [79]. The Closet Probability and Feature Grid (CPFG)-SLAM [80] is inspired by both ICP and NDT, searching for the nearest-neighbor grid instead of the nearest-neighbor point, and can achieve a more efficient registration of the point cloud in the off-road scenario.

Other registration methods include the Random Sample Consensus (RANSAC) algorithm-based registration method [81], which randomly chooses minimal points from each scan and then calculates the transformation, and the transformation with the largest number of inliers is selected and returned. Its time complexity depends on the subset size, the inlier ratio, and the number of data points; thus, its runtime can be prohibitively high in some cases. The Implicit Moving Least-Squares (IMLS) method leverages a novel sampling strategy and an implicit surface matching method, and demonstrates excellent matching accuracy, but is hard to operate in real time [82]. The Surface Element (Surfel) method [83,84,85] can represent a large-scale environment while maintaining dense geometric information at the same time. MULLS-ICP categorizes points into ground, facade, pillar, and beam, which are then registered by the multimetric ICP [86].

Feature-based methods extract relevant features from the point clouds, and then use them for successive pose estimation. Since these methods only use a selected part of the point cloud, they can be treated as “sparse” methods. Features are the points with distinct geometry within a locality [87]. Feature-based methods generally consist of three main phases: key point detection, feature description, and matching [88]. Summary and evaluation of the 3D feature descriptors can be found in [87,88,89]. Several feature descriptors—including the spinning images (SI) [90], the Fast Point Feature Histograms (FPFHs) [91], the Shape Context (SC) [92,93], and the Signature of Histograms of Orientations (SHOT) [94,95]—are applied for point cloud registration and loop-closure detection [60,93,95,96]. According to [97], SHOT is the descriptor that can give the fastest and most accurate results in the test. Feature descriptor methods are often employed in initial transformation calculations or loop-closure detection problems.

The state-of-the-art feature-based method LiDAR Odometry And Mapping (LOAM) has held first place in the KITTI odometry benchmark since it was first introduced in [98]. LOAM achieves both low drift and low computational complexity by running two algorithms in parallel. Feature points are selected as edge points with low smoothness and planar points with high smoothness, and then the transformation between consecutive scans is found by minimizing the point-to-edge distance for edge points and the point-to-plane distance for planar points, using the Levenberg–Marquardt (L–M) method. Inspired by LOAM, several methods have been proposed, including LeGO-LOAM [99], which first segments the raw point cloud using a range image, and then extracts features via a similar process to that used in LOAM, and performs a two-step L–M optimization; that is, [$t_{x},t_{y}$, $\theta_{yaw}$] are estimated using the edge features, while [$t_{z}$, $\theta_{roll},$ $\theta_{pitch}$] are estimated using the planar features. A summative table of representative LiDAR odometry methods is shown in Table 2.

In terms of currently available solutions, SLAMTEC offers user-friendly software [100] for robotic odometry, mapping, and control, with low-cost LiDAR. Livox offers products and packages for odometry and mapping. Cartographer is a widely used package for odometry and mapping with LiDAR.

**Table 2:** Summary of representative LiDAR odometry (LO).

Category	Method	Loop-Closure Detection	Accuracy ^1^	Runtime ^1^
Scan-matching	ICP [70]	No	Medium	High
	NDT [76]	No	Medium	High
	GMM [77]	No	Medium	-
	IMLS [82]	No	High	High
	MULLS [86]	Yes	High	Medium
	Surfel-based [83]	Yes	Medium	Medium
	DLO [101]	No	High	Low
	ELO [102]	No	High	Low
Feature-based	Feature descriptor [97]	No	Low	High
	LOAM [98]	No	High	Medium
	LeGO-LOAM [99]	No	High	Low
	SA-LOAM [103]	Yes	High	Medium

^1^ Adopted from [58,104].

### 2.4. Millimeter Wave (MMW) Radar

Radar stands for radio detection and ranging, which is another type of rangefinder. It is based on the emission and detection of electromagnetic waves in the radio frequency ranging from 3 MHz to 300 GHz (with wavelengths from 100 m to 1 mm). The radar equation (Equation (11)) depicts how the expected received power $p_{r}$ is a function of the transmitted power $p_{t}$, the antenna gain G, and the wavelength λ, as well as the Radar Cross-Section (RCS) σ and the range r from the target. Compared with its counterpart LiDAR, radar has superior detection performance under extreme weather conditions, since waves within this spectrum have weak interaction with dust, fog, rain, and snow. The Millimeter Wave (MMW) spectrum ranges from 30 GHz to 300 GHz (with wavelengths from 10 mm to 1 mm), which provides wide bandwidth and narrow beams for sensing, thus allowing finer resolution [105,106].

(11) $$p_{r}=\frac{p_{t}G^{2}\lambda^{2}\sigma }{{\left(4\pi \right)}^{3}r^{4}}$$

**Figure 4:**
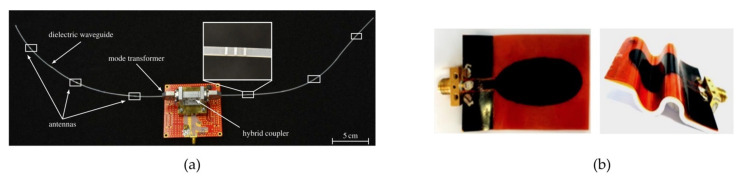
(**a**) HDPE as a dielectric waveguide for distributed radar antennas, reprinted with permission from [108]. Copyright IEEE 2019. (**b**) PANI/MWCNT fabricated antenna on a Kapton substrate, demonstrating good flexibility; reprinted with permission from [109]. Copyright John Wiley and Sons 2018.

In terms of polymer utilization in radar, Liquid Crystal Polymer (LCP) is regarded as a promising candidate as a substrate for MMW applications due to its flexibility, low dielectric loss, lower moisture absorption, and ability to withstand temperatures up to 300 °C [107]. The use of HDPE as a dielectric waveguide for distributed flexible antennas for proximity measurement in robotics applications is presented in [108] (Figure 4a). The use of conducting polymers such as polyaniline (PANI), doped with multiwalled carbon nanotubes, in the fabrication of antennas has demonstrated excellent flexibility and conformality in RF device manufacture [109] (Figure 4b).

**Figure 5:**
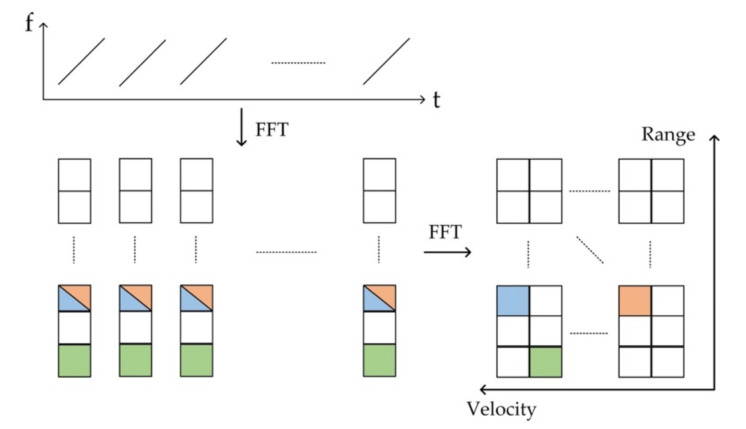
2D FFT of the beat frequency signal, redrawn from [115]. Copyright River Publishers 2017.

Based on the forms of the emitted wave, radar can be divided into pulsed and continuous-wave radar [110]; pulsed radar determines range based on the round-trip time of the electromagnetic wave, with its maximal detectable range and range resolution depending on its pulse cycle interval and pulse width, respectively. In contrast to pulsed radar, continuous-wave radar emits a continuous signal, and the most widely used waveform for robotics and automotive applications is Frequency-Modulated Continuous-Wave (FMCW), which can determine the range and velocity of the object simultaneously. The frequency of the emitted signal is modulated linearly against time, which is also referred to a chirp. The range and the velocity information are obtained by performing 2D Fast Fourier Transform (FFT) on the radar’s beat frequency signal [111,112,113], as demonstrated in Figure 5. Other waveforms for MMW radar are summarized in [111,114].

The angular location of detection should be discriminated so that the location of the objects can be resolved. Thanks to the short wavelength of MMW, the aperture size of radar antennas can be made small; hence, many antennas can be densely packed to form an array. With at least two receiver antennas, the Angle of Arrival (AoA) can be calculated from the measured phase difference at different receivers, which can be performed via 3D FFT [116]. A commonly used AoA measurement principle is called Multiple-Input–Multiple-Output (MIMO), which utilizes multiple transmitters and receivers to form an antenna array. Spaced real and virtual receivers can thus calculate the elevation and azimuth angle based on the phase shift in the corresponding directions. The virtue of a fixed antenna array is that the examined region is captured instantaneously; hence, no distortion will appear due to sensor movement and, thus, most automotive radar adopts this configuration. In addition to MIMO, the AoA can also be measured by a spinning directive antenna, for each moment the radar outputs an 1D power–range spectrum for the inspected line of sight, where the azimuth angle is the radar’s own azimuth angle relative to the fixed coordinate [115]. In [117,118], a designated spinning radar device called PELICAN was constructed and evaluated for mobile robotics applications.

An overview of MMW radar applications in robotics and autonomous vehicles can be found in [119,120]. Radar-based odometry methods can be classified into direct and indirect methods [121,122]; similar to LO, indirect methods involve feature extraction and association, whereas direct methods forego these procedures. Among the direct methods, in [123], the Fourier–Mellin Transform (FMT) is used for registering radar images in sequence, which relies on the translational and rotational properties of the Fourier transformation [124]. Similarly, FMT is also leveraged in [125], where the rotation and translation are calculated in a decoupled fashion, and a local graph optimization process is included. Since Doppler radar can measure radial velocity directly, the relative velocity of a stationary target is equal to the inverse sensor velocity. This method is implemented in [126,127,128,129], where a RANSAC algorithm is invoked for non-stationary outlier removal. Meanwhile, in [127], the 2D point-to-point ICP is used to obtain finer odometry results. Since Doppler measurement cannot survey rotational ego-motion directly, a hybrid method is used in [130] for angular rate measurement.

Among the indirect methods, some classical work in SLAM was carried out with the aid of MMW radar, including [131], which incorporated it with a Kalman filter framework operating in an environment with well-separated reflective beacons. The authors of [132] conducted a series of thorough works on mobile robot localization with MMW radar. In their work, new feature-extraction algorithms—Target Presence Probability (TPP) and a confidential model—showed superior performance compared with constant thresholding and Constant False Alarm Rate (CFAR) [133]. Since radar detection can be impaired by false alarms and clutter, feature association may become problematic; thus, the feature measurements would be better modeled as Random Finite Sets (RFSs) with arbitrary numbers of measurements and orders, and incorporated with the RB (Rao–Blackwellized)-PHD (Probability Hypothesis Density) filter [134,135] for map building and odometry.

Some more recent works include [136], which extracts the Binary Annular Statistics Descriptor (BASD) for feature matching, and then performs graph optimization; and [137], where SURF and M2DP [96] descriptors are computed from radar point clouds for feature association and loop-closure detection, respectively; as well as the use of SIFT in [138]. Radar measurements are noisy and, thus, may worsen the performance of scan-matching algorithms used for LiDAR, such as ICP and NDT; nevertheless, G-ICP [70] showed good validity in [139], where the covariance of each measurement was assigned according to its range; the same can be said of NDT in [140] and GMM in [141], which incorporated detection clustering algorithms including k-means, DBSCAN, and OPTICS. In [142], Radar Cross-Section (RCS) was used as a cue for assisting with feature extraction and Correlative Scan Matching (CSM). The authors of [143,144] devised a new feature-extraction algorithm for MMW radar power–range spectra, plus a multipath reflection removal process, and then performed data association via graph matching.

Ref. [145] analyzed radar panoramic image distortion caused by vehicle movement and the Doppler effect in detail. CFAR detection was used for feature extraction, and then feature association and optimization were applied to retrieve both the linear velocity and the angular rate of the mobile platform. The method in [146] was based on a similar assumption, but was applied in a more elegant way, utilizing the feature-extraction algorithm proposed in [143], and calculating ORB descriptors for feature association. These methods demonstrated better results compared with the FMT-based methods, but their performance may deteriorate when the robot is accelerating or decelerating [122]. A summative table of representative radar odometry methods is shown in Table 3.

In terms of currently available solutions, NAVTECH offers radar devices that have been widely used in radar odometry research. Yeti [146] is a package that works with NAVTECH radar that removes motion distortion and provides odometry data.

### 2.5. Vision

Cameras can acquire visual images for various applications. Various cameras—including monocular cameras, stereo cameras, RGB-D cameras, event cameras, and omnidirectional cameras—are employed for robotic tasks [148]. Solid-state image sensors, including Charge-Coupled Devices (CCDs) and Complementary Metal-Oxide Semiconductors (CMOSs), are the basis of the cameras used for imaging [149]. Photodetectors take the role of converting electromagnetic radiation into an electronic signal that is ideally proportional to the incident light, and are mostly fabricated from semiconductors made from materials such as Si, GaAs, InSb, InAs, and PbSe. When a photon is absorbed, it creates a charge-carrier pair, and the movement of charge carriers produces a photocurrent to be detected. Compared with inorganic photodetectors, organic photodetectors exhibit attractive properties such as flexibility, light weight, and semi-transparency. Heterojunction diodes based on polymers such as P3HT:PC_71_BM, PCDTBT:PC_71_BM, and P3HT:PC_61_BM as the photon active layer are fabricated [150]. Organic photodetectors can be classified into organic photoconductors, organic photon transistors, organic photomultiplication devices, and organic photodiodes. These organic photodetectors demonstrate utility in imaging sensors [151]. As a recent topic of interest, narrowband photodetectors can be fabricated from materials with narrow bandgaps, exhibiting high selectivity of wavelength [152].

Several camera models can describe the projection of 3D points onto the 2D image plane. The most common is the pinhole model (Equation (12)), which is widely applied for monocular and stereo cameras, projecting a world point P to its image coordinate $P^{\prime }$, where α and β are coefficients of focal length and pixel length, respectively, while $c_{x}$ and $c_{y}$ describe the coordinates of the image’s center [153]. Other models—including the polynomial distortion model, the two-plane model, and the catadioptric camera model—are summarized in [154,155].

(12) $$P^{\prime }=\left[\begin{array}{c} x^{\prime }\\ y^{\prime }\\ z \end{array}\right]=\left[\begin{array}{cccc} \alpha & 0 & c_{x} & 0\\ 0 & \beta & c_{y} & 0\\ 0 & 0 & 1 & 0 \end{array}\right]\left[\begin{array}{c} x\\ y\\ z\\ 1 \end{array}\right]=\left[\begin{array}{cccc} \alpha & 0 & c_{x} & 0\\ 0 & \beta & c_{y} & 0\\ 0 & 0 & 1 & 0 \end{array}\right]P$$

The term Visual Odometry (VO) was first coined by [156]. As suggested by its name, motion estimation of the mobile platform is performed solely from visual input. With recent advancements in the VO systems with loop-closure, the boundary between VO and Visual-SLAM (V-SLAM) has become blurred; nevertheless, VO systems devote more attention to ego-motion estimation than to map building [157]. There exist several review papers on the VO systems—[13,158,159,160] provided a comprehensive overview of VO and V-SLAM; the two-part survey [161,162] highlighted feature-based VO; while [163,164,165,166] conducted reviews of recent advancements in VO and V-SLAM using state-of-the-art data-driven methods. VO can be classified into geometry-based methods and learning-based methods; geometry-based methods can be further categorized into feature-based approaches, appearance-based approaches, and hybrid approaches.

Geometry-based methods explicitly model camera pose based on multi-view geometry; among them, feature-based approaches are currently the most prominent solution for VO. Features denote salient image structures that differ from their neighbors, and they need to be first extracted and then located by feature detectors, such as Harris, Shi–Tomasi, FAST, or MSER. To match features between different images, they need to be described by the adjacent supported region with feature descriptors, such as ORB [167], BRIEF [168], BRISK, or FREAK. Some algorithms, such as SIFT [169] and SURF [170], involve both a detector and a descriptor. For an in-depth survey of feature detectors and descriptors, please refer to [171,172]. Based on the pose estimation solver, feature-based approaches can be further decomposed into 2D-to-2D methods, 3D-to-2D methods, and 3D-to-3D methods [161].

2D-to-2D methods are formulated from the so-called epipolar geometry; the motion of the camera is resolved by calculating the essential matrix E, which encapsulates the translational parameters t and the rotational parameters R of the camera motion; p and $p^{\prime }$ are corresponding image points.

(13) $$p^{T}Ep^{\prime }=0$$

(14) $$E=\left[t_{\times }\right]R.$$

In terms of the minimal sets of point correspondences required to generate a motion hypothesis, several n-point algorithms were proposed, with a tutorial to be found in [173], including the eight-point algorithm [174]—a linear solver with a unique solution, which is implemented in the monocular version of [175] and LiBVISO2 [176]. The seven-point algorithm applies the rank-constraint of the essential matrix [177], and is more efficient in the presence of outliers, but there may exist three solutions, and all three must be tested. The six-point algorithm further imposes the trace-constraint of the essential matrix, and may return either a single solution [178] or up to six solutions [179]; the former cannot perform in the presence of a planar scene [180]. The six-point algorithm may also serve as a minimal solver for a partially calibrated camera with unknown focal length [181,182]. If the depth of an object is unknown, only five parameters are calculated for camera motion (two for translation and three for rotation); hence, a minimal set of five correspondences is adequate. The five-point algorithm [182,183,184,185,186,187] solves a multivariate polynomial system and returns up to 10 solutions; this system can be solved via a Groebner basis [185], hidden variable approach [182], PolyEig [187], or QuEst [186]. It has the ability to work under planar scenes, and also quadric surfaces [188]. If the rotational angle can be read from other sensors, such as IMUs, then the four-point algorithm [189] and the three-point algorithm [190,191] can be used. By imposing the non-holonomic constraint of the ground vehicle, the one-point algorithm was proposed in [192]; however, the camera needed to be mounted above the rear axis of the vehicle. For cameras moving in a plane, the two-point algorithm was proposed [193,194]. Note that the above algorithms usually incorporate the RANSAC framework, indicating that fewer points lead to faster convergence.

The 3D-to-2D methods aim at recovering camera position and orientation from a set of correspondences between the 3D points and their 2D projections, which is also known as the Perspective-n-Point (PnP) problem. Several PnP algorithms were summarized in a recent review paper [195]. Three points is the minimal set to solve the problem; [196] covered the early solutions to the problem, in which the covered solutions suffered from instability stemming from the unstable geometric structure [197]. The first complete analytical solution to the P3P problem was given in [198]. An improved triangulation based method Lambda Twist P3P was proposed in [199]. Ref. [200] directly computed the absolute position and orientation of the camera as a function of image coordinates and their world-frame coordinates instead of employing triangulation, and demonstrated better computational efficiency. This was further improved by [201]. Nonetheless, it is desirable to incorporate larger point sets to bring redundancy and immunity to noise. For more general cases where n >3, PnP solutions based on iterative and non-iterative methods have been proposed. Iterative PnP solutions—including LHM [202] and PPnP [203]—are sensitive to initialization, and may get stuck in local minima. SQPnP [204] casts PnP as a Quadratically Constrained Quadratic Program (QCQP) problem, and attains global optima. Among the non-iterative methods, the first efficient algorithm EPnP was presented in [205], which allocates four weighted virtual control points for the whole set of points to improve efficiency. To improve accuracy when the point set is small (n ≤5), RPnP [206] was proposed, which partitions n points into (n−2) sets, and forms a polynomial system to determine the intermediate rotational axis; the rotational angle and translational parameters are retrieved by performing Singular Value Decomposition (SVD) of a linear equation system. This was further refined by the SRPnP algorithm [207]. The Direct-Least-Squares (DLS) method [208] employs a Macaulay matrix to find all roots of the polynomials parameterized from Carley parameterization, which can guarantee global optima, but suffers from degeneration for any 180° rotation. This issue was circumvented by OPnP [209], which also guarantees global optima using non-unit quaternion parameterization.

The 3D-to-3D methods recover transformation based on sets of points with 3D information, which is similar to the case for LiDAR point cloud registration. Generally, 3D data acquired with a stereo camera or RGB-D camera are noisier than those acquired by LiDAR; thus, the performance of 3D-to-3D methods is usually inferior to that of the 3D-to-2D methods. Similar approaches to those adopted in LiDAR systems—such as ICP [210], NDT [211], and feature registration [91,212]—have been applied for visual odometry, with surveys and evaluations of their performance to be found in [213,214].

Appearance-based approaches forego the feature-matching step and use the pixel intensities from the consecutive images instead; consequently, they are more robust under textureless environments. They can be further partitioned into regional-matching-based and optical-flow-based methods. The regional-matching-based methods recover the transformation by minimizing the photometric error function, and have been implemented for stereo cameras [215,216,217,218], RGB-D cameras [219], and monocular cameras in dense [220], semi-dense [221], and sparse [222,223] fashion. Optical-flow-based methods retrieve camera motion from the point velocity measured on the image plane, as the apparent velocity of a point $X\in {\mathbb{R}}^{3}$ results from the camera linear velocity v and angular velocity ω, where ${\left[\omega \right]}_{\times }$ stands for the skew-symmetric matrix with the vector $\omega \in {\mathbb{R}}^{3}$:

(15) $$\dot{X}={\left[\omega \right]}_{\times }X+v$$

Commonly used algorithms for optical flow field computation include the Lucas–Kanade algorithm and the Horn–Schunck algorithm. Most optical-flow-based methods are derived from the Longuet–Higgins model and Prazdny’s motion field model [224]; for an overview, please refer to [225].

Hybrid approaches combine the virtues of robustness from feature-based approaches and abundance in information of appearance-based approaches. SVO [226] utilizes direct photometric error minimization for incremental motion estimation, followed by feature matching for pose refinement. Ref. [227] leveraged direct tracking adopted from LSD-SLAM [216] for inter-keyframe pose tracking and feature-based tracking for incremental motion estimation, which also served as a form of motion prior to keyframe refinement. A similar notion was adopted and improved upon in [228]. Conversely, in [229] the direct module from DSO [222] was used for real-time camera tracking, and the feature-based module from ORB-SLAM [157] was used for globally consistent pose refinement. Meanwhile, in [230], the geometric residual and the photometric residual were optimized jointly for each frame. In terms of currently available solutions, the RealSense T265 depth camera offers a standalone solution to directly output odometry [231]. A summative table of representative visual odometry methods is shown in Table 4.

### 2.6. Discussion

Polymers have been employed in various sensors that are applicable for odometry, as summarized in Table 5. The pronounced virtues of using polymers are flexibility, light weight, and low cost.

Sensor modalities are the most dominant drivers for the evolution of odometry methods; generally, a new wave of odometry methods emerges every time new applicable sensors appear. Event-based cameras are bio-inspired sensors; unlike conventional cameras, which image the whole scene at a fixed rate, event cameras respond to changes in brightness at the individual pixel level, have the merits of high temporal resolution (i.e., microseconds), high dynamic range, and low latency [236,237]. Odometry methods based on event cameras were proposed in [238]. Doppler LiDAR has the ability of long-range detection and radial velocity measurement; hence, it endows mobile platforms with better sensing capacity [239,240]. An MEMS Focal Plane Switch Array (FPSA) with wide FOV was recently proposed in [241], achieving better performance than current OPA LiDAR, and suitable for mass production in CMOS foundries.

Learning-based methods have recently attracted much attention and come to the fore, as they do not rely on handcrafted algorithms based on physical or geometric theories [9], and demonstrate comparable or even better performance than traditional methods. To enumerate some, for visual odometry, DeepVO [242] leverages deep Recurrent Convolutional Neural Networks (RCNNs) to estimate pose in an end-to-end fashion; D3VO [243] formulates the deep prediction of depth, pose, and uncertainty into direct visual odometry. For inertial navigation, ref. [244] introduced a learning method for gyro denoising, and estimated attitudes in real-time. For radar odometry, ref. [245] presented an unsupervised deep learning feature network for subsequent pose estimation. For LiDAR odometry, LO-NET [246] performs sequential learning of normal estimation, mask prediction, and pose regression. However, learning-based methods may deteriorate at previously unseen scenes.

Each sensor has its strengths and weaknesses, as summarized in Table 6, which reveals that there is no single sensor that can handle all conditions, while one sensor may complement another in at least one aspect. Thus, a multisensor fusion strategy is favored.

## 3. Sensor Fusion

There is no single sensor that can perform all measurements; thus, stitching data from various sensors for complement and verification is more desirable. Generally, sensor fusion is used for two purposes: redundancy and complement. Redundancy is provided by sensors with the same measurement capability (e.g., range measurements from LiDAR and radar), and its aim is to improve the accuracy of the measurements. Complement is provided by sensors with diverse measurement capabilities (e.g., range measurement from LiDAR and speed measurement from radar), and its aim is to enrich the collected information [248]. As a rule of thumb, measurements fused from two low-end sensors can attain similar or better results than those from a single high-end sensor, since it is mathematically proven that the covariance of two measurements is lower than their individual variances [249]. One commonly used categorization of sensor fusion is based on the input to the fusion framework: For low-level fusion, raw sensor data are directly fed into the fusion network. Medium-level fusion takes place where features are first extracted from the raw data and then fused together. High-level fusion, also called decision fusion, combines decisions from individual systems [250]. For mobile robot odometry—especially for Visual–Inertial Odometry (VIO)—two main approaches are used for sensor fusion, namely, the tightly coupled approach and the loosely coupled approach. In the loosely coupled approach, each sensor has its own estimator (e.g., VO and IMU), and the final result is a combination of each estimator, while in the tightly coupled approach the sensor measurements are directly fused in a single processor [166].

The taxonomy of sensor fusion methods for mobile robot odometry is dependent on their working principle, and adopted from recent surveys on sensor fusion [10,248,251,252]. Filter-based methods [253] and optimization-based methods [254,255] are summarized below.

### 3.1. Filter-Based

#### 3.1.1. Probability-Theory-Based

Probability-based methods represent sensor data uncertainty as a Probability Density Function (PDF); data fusion is built upon the Bayesian estimator, given a measurement set $Z^{k}=\left\{z_{1},\ldots ,z_{k}\right\}$ and the prior distribution $p\left(x_{k}|Z^{k-1}\right)$, and the posterior distribution of the estimated state $x_{k}$ at time *k* is given by:

(16) $$p\left(x_{k}|Z^{k}\right)=\frac{p\left(z_{k}|x_{k}\right)p\left(x_{k}|Z^{k-1}\right)}{p\left(Z^{k}|Z^{k-1}\right)}$$

The well-known Kalman Filter (KF) is an analytical solution to the Bayes filter, and is probably the most popular method for sensor fusion. The standard KF has two steps: the prediction step, and the correction step. In the prediction step, the predicted state mean ${\bar{\mu }}_{t}\;$ and covariance ${\bar{\sum }}_{t}$ are calculated as follows:

(17) $${\bar{\mu }}_{t}=A_{t}\mu_{t-1}+B_{t}u_{t}$$

(18) $${\bar{\sum }}_{t}=A_{t}\Sigma_{t-1}A_{t}^{T}+R_{t},$$

where $A_{t}$ and $B_{t}$ are the state and control transition matrices, respectively, $R_{t}$ is the covariance matrix of motion noise, $u_{t}$ is the control vector, and the indices *t* and *t* − 1 represent the current and previous timestamp, respectively.

In the correction step, the Kalman gain $K_{t}$, as well as the updated state mean $\mu_{t}$ and the covariance $\sum_{t}$, are calculated as follows:

(19) $$K_{t}={\;\bar{\sum }}_{t}C_{t}^{T}{\left(C_{t}{\;\bar{\sum }}_{t}C_{t}^{T}+Q_{t}\right)}^{-1}$$

(20) $$\mu_{t}={\bar{\mu }}_{t}+K_{t}\left(z_{t}-C_{t}{\bar{\mu }}_{t}\right)$$

(21) $$\sum_{t}=\left(I-K_{t}C_{t}\right){\;\bar{\sum }}_{t},$$

where $C_{t}$ is the measurement matrix, and $Q_{t}$ is the covariance matrix of measurement noises. For a full derivation of the KF, please refer to [256].

The standard KF requires both the motion and measurement models to be linear; for nonlinear systems, the Extended Kalman Filter (EKF) and Unscented Kalman Filter (UKF) can be adopted, which are based on first- and second-order Taylor expansion of current estimation, respectively. The EKF has been implemented for Visual–Inertial Odometry (VIO) [257], LiDAR–Inertial Odometry (LIO) [258], and Radar–Inertial Odometry (RIO) [128]; an error state space propagation strategy is usually adopted due to its superior properties [259]. Inertial measurement serves to propagate the filter state when the measurements from the camera or LiDAR are incorporated into the filter update. Variants of the EKF, such as the UKF [260], Multi-State Constraint Kalman Filter (MSCKF) [261,262]—which incorporates poses of past frames to marginalize features from the state space. Iterated EKF [263], Cubature Kalman Filter (CKF) [264], fuzzy logic KF [265], covariance intersection KF [266] and invariant EKF based on the Lie group [267], have been proposed in the literature.

Although widely implemented, the KF and its variants can handle nonlinearity only to a limited degree. Monte Carlo (MC)-simulation-based methods express the underlying state space as weighted samples, and do not make assumptions of the underlying probability distribution. Markov Chain Monte Carlo (MCMC) and Sequential Monte Carlo (SMC) are two types of MC [268]; SMC (also known as Particle Filter) is more frequently seen in robot odometry. Particle Filter (PF) uses a set of particles $x_{t}^{\left[m\right]}$ with index m at time t to resemble the real state space; the transition of particles takes place according to the state transition function, and then the weight of each sample is calculated as follows:

(22) $$w_{t}^{\left[m\right]}\propto p\left(z_{t}|x_{t}^{\left[m\right]}\right)$$

The real trick of PF is the importance sampling step, where the samples are resampled according to their weight; this step approximates the posterior distribution of the Bayes filter [269]. The Rao–Blackwellized Particle Filter (RBPF) is one of the most importantimplementations of PF for odometry, introduced in [270] and refined in [271]. It is built upon the Rao–Blackwell theorem, which states the fact that sampling of $x_{1}$ from the distribution $p\left(x_{1}\right)$, and then $x_{2}$ conditioned on $x_{1}$ from $p\left(x_{2}\right)$, will do no worse than sampling from their joint distribution $p\left(x_{1},x_{2}\right)$. Some notable recent advancements of PF include Particle Swarm Optimization (PSO)-aided PF [272], entropy-weighted PF [273], Inter-Particle Map Sharing (IPMS)-RBPF [274], and a 6D translation–rotation decoupled RBPF with an autoencoder network to output hypotheses of rotation [275]. The general trend of PF is to either reduce the sampling size via a better sampling strategy or simplify the map representation.

#### 3.1.2. Evidential-Reasoning-Based

Evidential-reasoning-based methods rely on the Dempster–Shafer (D–S) theory [276], combining evidence from several sources to prove a certain hypothesis, which can be seen as a generalization of the Bayes theory when ignorance about the hypothesis reaches zero. For a given problem, let X be a finite set of all possible states of the system (also called the Frame of Discernment (FOD)), where the power set $2^{X}$ represents all possible subsets of X. D–S assigns a mass $m\left(E\right)$ to each element E of $2^{X}$, representing the proportion of available evidence that supports the system state x belonging to E, and the mass function has the following properties (∅ stands for empty set):

(23) $$m\left(\emptyset \right)=0\;\;\underset{E\in 2^{X}}{\sum }m\left(E\right)=1$$

Using m, the probability interval $bel\left(E\right)⩽P\left(E\right)⩽pl\left(E\right)$ can be obtained, where the lower bound belief is defined as $bel\left(E\right)=\sum_{B\subseteq E}m\left(B\right)$ and the upper bound’s plausibility is defined as $pl\left(E\right)=\sum_{B\cap E\neq ⲫ}m\left(B\right)$. Evidence from two sources is combined via the D–S combination rule as follows:

(24) $$m_{1}⊕m_{2}\left(E\right)=\frac{\sum_{A\cap B=E}m_{1}\left(A\right)m_{2}\left(B\right)}{1-\sum_{A\cap B=\emptyset }m_{1}\left(A\right)m_{2}\left(B\right)}$$

The notion of D–S has been deployed for sensor fusion in odometry—as demonstrated in [277,278]—and map building in [279]. Its main advantage is that it requires no prior knowledge of the probability distribution, and has proven utility for systems in which one sensor reading is likely to be unreliable or unavailable during operation.

#### 3.1.3. Random-Finite-Set-Based

Random Finite Set (RFS)-based methods treat the system state as a finite-set-valued random variable instead of a random vector, and the size (cardinality) of the set is also a random variable. RFS is deemed to be an ideal extension of the Bayes filter, and has been widely implemented for Multi-Target Tracking (MTT). Propagating the whole RFS Probability Density Function (PDF) is computationally intractable; thus, the first statistical moment of RFS—Probability Hypothesis Density (PHD)—is used to construct the PHD filter, as it is for the Kalman filter (i.e., mean and covariance) [134,280]. With the PHD filter, the target state is modelled as the union (∪) of different RFSs:

(25) $$X_{k}=[\underset{\zeta \in X_{k-1}}{\cup }S_{k|k-1}\left(\zeta \right)]\cup \Gamma_{k}$$

where $S_{k|k-1}\left(\zeta \right)$ represents the surviving targets from time k−1, and is modelled as a Bernoulli RFS, which means that it either survives—with probability $P_{S,k}\left(X_{k-1}\right)$—to take the new value $X_{k}$, or dies—with probability $1-P_{S,k}\left(X_{k-1}\right)$—into the empty set ∅. $\Gamma_{k}$ represents spontaneously born targets at time k, and is modelled as a Poisson RFS with intensity (PHD) $\gamma_{k}\left(\cdot \right)$. The observation state is also modelled as the union of different RFSs:

(26) $$Z_{k}=[\underset{x\in X_{k}}{\cup }\Theta_{k}\left(x\right)]\cup K_{k}$$

where $\Theta_{k}\left(x\right)$ represents detected targets, and is modelled as a Bernoulli RFS, which means that it is either detected—with probability $P_{D,k}\left(X_{k}\right)$ yielding $Z_{k}$ by the observation function $g_{k}\left(Z_{k}|X_{k}\right)$—ormissed—with probability $1-P_{D,k}\left(X_{k}\right)$—into the empty set ∅. $K_{k}$ represents false alarms, and is modelled as a Poisson RFS with intensity (PHD) $\kappa_{k}\left(\cdot \right)$. The prediction and update equation of the PHD filter are as follows, where $D_{k}$ is the first moment (i.e., PHD) of the state RFS:

(27) $$D_{k|k-1}\left(X_{k}\right)=\int P_{S,k}\left(X_{k-1}\right)f_{k|k-1}\left(X_{k}|X_{k-1}\right)D_{k-1}\left(X_{k-1}\right)dX_{k-1}+\gamma_{k}\left(X_{k}\right)$$

(28) $$D_{k}\left(X_{k}\right)=\left(1-P_{D,k}\left(X_{k}\right)\right)D_{k|k-1}\left(X_{k}\right)+\underset{z\in Z_{k}}{\sum }\frac{P_{D,k}\left(X_{k}\right)g_{k}\left(z_{i}|X_{k}\right)D_{k|k-1}\left(X_{k}\right)}{\kappa_{k}\left(z_{i}\right)+\int P_{D,k}g_{k}\left(z_{i}|\zeta \right)D_{k|k-1}\left(\zeta \right)d\zeta }$$

The PHD is not a PDF, and is not necessarily integrated into one, integrating the PHD will yield the number of targets in the space [281,282]. The PHD filter is handy when modelling phenomena such as object block, missing detection, or false alarms. Thus, it has been used for Radar Odometry (RO), since both misdetection and false feature association can be commonly encountered in RO [132]. A comparison between PHD filters and state-of-the-art EKFs [267] was conducted in [283], demonstrating robustness of the PHD filter. A C (Cardinalized)-PHD filter propagates the distribution of the number of targets (cardinality) in addition to the PHD function; this auxiliary information enables higher modelling accuracy [284]. Alternatively, a multi-Bernoulli filter was implemented in [285,286].

### 3.2. Optimization-Based

Optimization-based (smoothing) methods correspond to estimating the full state given the whole observations up to current moment. This is the so-called full-SLAM solution. An intuitive way to achieve this is via the factor graph method, which builds a graph whose vertices encode robot poses and feature locations, with edges encoding the constraints between vertices arising from measurements [255,287]. This is cast into an optimization problem that minimizes $F\left(x\right)$:

(29) $$x^{*}=\underset{x}{\mathrm{argmin}}\;F\left(x\right)$$

(30) $$F\left(x\right)=\underset{\langle ij\rangle \in C}{\sum }e_{ij}^{⊤}\Omega_{ij}e_{ij}$$

where C stands for the set index pair between nodes, $\Omega_{ij}$ stands for the information matrix between nodes i and j, and $e_{ij}$ is the error function modelling the error between expected and measured spatial constraint. Some generic solvers—including GTSAM, g^2^o, Ceres, SLAM++, and SE-Sync—are frameworks approaching nonlinear optimization problems, and also provide solutions for SLAM [288]. g^2^o is a library of general graph optimization algorithms. It contains predefined types of nodes and edges to simplify the structure of SLAM algorithms, and to construct SLAM algorithms quickly. GTSAM is an optimizer for maximizing posterior probabilities using factor graphs and Bayes networks, and is commonly used as a C++ library for smoothing and mapping, as well as being widely used in SLAM and computer vision. Ceres Solver is a library developed by Google for nonlinear optimization, and is heavily used in Google’s open-source LiDAR SLAM project Cartographer. Its process is simple and well understood, with a variety of built-in solvers for a wide range of application scenarios, and can solve common data-fitting, least-squares, and nonlinear least-squares problems. A summative table of representative fusion methods is shown in Table 7.

### 3.3. Discussion

Sensor fusion at high levels has better scalability and ease of modification to incorporate more sensors, while it generally shows better accuracy at low levels (i.e., the raw-data level); thus, tightly coupled fusion has become a trend in recently proposed methods. However, traditional methods need accurate models and careful calibration, so employing machine learning for sensor fusion in odometry has also become an open research topic. In [299], sequences of CNNs were used to extract features and determine pose from a camera and 2D LiDAR. Some learning-based methods, such as VINet and DeepVIO [300], demonstrate comparable or even better performance than traditional methods.

## 4. Future Prospects

### 4.1. Embedded Sensors for Soft Robots’ State Estimation and Perception

Soft embedded sensors have been employed for soft robots in strain, stress, and tactile sensing [301]. However, soft sensors generally exhibit nonlinearity, hysteresis, and slow response. To overcome these issues, multisensor fusion strategies for soft sensors—such as [302,303]—have been proposed. Recent achievements have also brought soft sensors and machine learning techniques together for robot kinematic estimation [304].

The need for soft robots to explore unstructured environments is also growing [305]. For example, the Visual Odometry (VO) method was used in a soft endoscopic capsule robot for location tracking [306]. However, current implantations still mostly rely on solid-state sensors, and employing soft sensors could greatly improve the flexibility and compatibility of these systems.

### 4.2. Swarm Odometry

Multiple robots can perform tasks more quickly and are more robust where single agents may fail [307]. This has been implemented in homogeneous systems, either in a centralized [308] or a decentralized [309] fashion, where multiple drones self-estimate their state from onboard sensors, and communicate with the base station or one another. This may also be deployed in a heterogeneous system, where the UAVs and UGVs work together. However, the scalability of the system, the relative pose estimation between agents, the uncertainty of the relative pose estimation, and the limited communication range still challenge such research.

### 4.3. Accelerating Processing Speed at the Hardware Level

While most current solutions rely on complex and time-consuming operations at the software level, this can be alleviated by using FPGAs (Field-Programmable Gate Arrays) or other dedicated processors [310] to integrate sensors and odometry algorithms at the hardware level, which enables odometry estimation at very high rates. This is expected to save computational resources and improve the real-time performance of the odometry systems.

## 5. Conclusions

In this paper, a comprehensive review of odometry methods for indoor navigation is presented, with detailed analysis of the state estimation algorithms for various sensors, including inertial measurement sensors, visual cameras, LiDAR, and radar, with an investigation of the applications of polymeric materials in those sensors. The principles and implementation of sensor fusion algorithms that have been successively deployed in indoor odometry are also reviewed. Generally, polymers introduce flexibility and compatibility to the sensors, and reduce the cost of their mass production. They may also serve as embedded solutions that enable novel applications of odometry technology, such as in soft endoscopic capsules.

Although mature solutions exist, the improvement of indoor odometry/localization accuracy is still an ongoing research topic. It is vital to achieve a sub-centimeter level for safe navigation. Prospective research areas within this topic including advanced sensor technology, algorithm enhancement, machine intelligence, human–robot interaction, information fusion, and overall performance improvement.

## Figures and Tables

**Table 1:** Summary of recent reviews on sensors and sensor fusion for SLAM and odometry.

Reference	Remarks
Bresson et al. [6] 2017	SLAM in autonomous driving
Mohamed et al. [7] 2019	Odometry systems
Huang et al. [8] 2020	Representative LiDAR and visual SLAM systems dictionary
Chen et al. [9] 2020 Fayyad et al. [10] 2020	Deep learning for localization and mapping
Yeong et al. [11] 2021	Sensor and sensor calibration methods for robot perception
El-Sheimy et al. [1] 2021	Overview of indoor navigation
Taheri et al. [12] 2021	Chronicles of SLAM from 1986–2019
Servières et al. [13] 2021	Visual and visual-inertial odometry and SLAM

**Table 3:** Summary of representative radar odometry (RO).

Category	Method	Automotive Radar (A)/Spinning Radar (S)	Radar Signal Representation	Loop-Closure
Direct methods	Fourier–Mellin transform [123]	S	Radar image	Yes
	Doppler-effect-based [126]	A	Point cloud	No
Indirect methods	Descriptor [137]	S	Radar image	Yes
	ICP [139]	A	Point cloud	No
	NDT [140]	Both	Point cloud	No
	GMM [141]	A	Point cloud	No
	Graph-matching [144]	S	Radar image	No
	Distortion resolver [145]	S	Radar image	No
Hybrid methods	RADARODO [130]	A	Radar image	No
	*SE(3)* RO [147]	A	Point cloud	No

**Table 4:** Summary of representative visual odometry (VO).

Category	Implementation	Camera Type	Loop-Closure	Remark
Feature-based	MonoSLAM [232]	Mono	No	
	PTAM [233]	Mono	No	5-point initiation
	S-PTAM [234]	Stereo	Yes	
	ORB-SLAM3 [157]	Mono/Stereo	Yes	PnP re-localization
Appearance-based	DTAM [220]	Mono	No	Dense
	LSD-SLAM [221]	Mono	Yes	Semi-dense
	DSO [222]	Mono	No	Sparse
Hybrid	SVO [226]	Mono	No	

**Table 5:** Summary of representative applications of polymers in sensors for odometry.

Sensor	Material	Major Role(s)	Merit(s) Comparing with Non-Polymeric Counterparts
Accelerometer	SU-8 [20]	Proof mass and flexure	Low Young’s modulus and high sensitivity
	Not reported [21]	Optical waveguide	High sensitivity
Gyroscope	PDMS [27]	Proof mass	Reduced driving force
	Not reported [26]	Optical waveguide	Low cost
LiDAR	Acrylate polymer [49]	Phase modulator and waveguide	High thermo-optic coefficients and low thermal conductivity
	P(VDF-TrFE) [51]	Actuator	Low cost
Radar	LCP [109]	Substrate	Low dielectric loss
	PANI [108]	Antenna	Flexibility and conformality
	HDPE [107]	Waveguide	Flexibility
Camera	MEHPPV:PCBM [235]	Photodetector	Wavelength tunability

**Table 6:** Comparison of onboard sensors for indoor odometry.

Sensor	Best Reported Accuracy		Cost	Advantages	Disadvantages
	Translation Error	Rotation Error (deg/m)			
IMU	0.97% ^1^	0.0023 ^1^	Low–high	Self-contained	Drift
LiDAR	0.55% ^2^	0.0013 ^2^	Medium–high	High accuracy; dense point cloud	Large volume; sensitive to weather
Radar	1.76% ^3^	0.005 ^3^	Medium–high	Weatherproof; radial velocity measurement	Sparse point cloud; high noise level
Camera	0.53% ^2^	0.0009 ^2^	Low–medium	Rich color information; compact	Sensitive to illumination; ambiguity in scale (monocular camera)

^1^ Adopted from [247]. ^2^ Adopted from the KITTI odometry benchmark. ^3^ Adopted from [245].

**Table 7:** Summary of representative multisensor fusion odometry methods.

Method			Implementation and Year	Loosely Coupled/Tightly Coupled	Sensor Suite ^1^	Loop-Closure
Filter-based	Probability-theory-based	Kalman filter	MSCKF [262], 2013	T	V-I	No
			ROVIO [257], 2015	T	V-I	No
			LINS [289], 2020	T	L-I	No
			FAST-LIO [258], 2020	T	L-I	No
			EKF RIO [128], 2020	T	R-I	No
			LVI-Odometry [290], 2018	L	V-L-I	No
			LIC fusion [291], 2019	T	V-L-I	No
		Particle filter	FastSLAM [292], 2002		L-O	No
	Evidential-reasoning-based	D–S combination	[277], 2013	L	GPS-I	No
	Random-finite set-based	PHD filter	PHD-SLAM 2.0 [283], 2021	T	L-O	No
Optimization-based			OKVIS [293], 2014	T	V-I	No
			VINS-MONO [294], 2018	T	V-I	Yes
			Kimera [295], 2020	T	V-I	Yes
			LIO-mapping [296], 2019	T	L-I	No
			LIO-SAM [297], 2020	T	L-I	Yes
			LVI-SAM [298], 2021	T	V-L-I	Yes

^1^ V: vision, L: LiDAR, R: radar, I: IMU, O: wheel odometer.

## Data Availability

Not applicable.
